# Predicting long-term sickness absence among employees with frequent sickness absence

**DOI:** 10.1007/s00420-018-1384-6

**Published:** 2018-11-24

**Authors:** Annette Notenbomer, Willem van Rhenen, Johan W. Groothoff, Corné A. M. Roelen

**Affiliations:** 1 Division Community and Occupational Medicine, Department of Health Sciences, University Medical Center Groningen, Rijksuniversiteit Groningen, Gezondheidswetenschappen, sectie Sociale Geneeskunde, Antonius Deusinglaan 1, 9713 AV Groningen, The Netherlands; 2 Center for Leadership and Management Development, Business University Nyenrode, Breukelen, The Netherlands; 3 Arbo Unie, Utrecht, The Netherlands

**Keywords:** Absenteeism, Sick leave, Prediction model, ROC analysis, Occupational health, Health surveillance

## Abstract

**Purpose:**

Frequent absentees are at risk of long-term sickness absence (SA). The aim of the study is to develop prediction models for long-term SA among frequent absentees.

**Methods:**

Data were obtained from 53,833 workers who participated in occupational health surveys in the period 2010–2013; 4204 of them were frequent absentees (i.e., employees with ≥ 3 SA spells in the year prior to the survey). The survey data of the frequent absentees were used to develop two prediction models: model 1 including job demands and job resources and model 2 including burnout and work engagement. Discrimination between frequent absentees with and without long-term SA during follow-up was assessed with the area under the receiver operating characteristic curve (AUC); (AUC) ≥ 0.75 was considered useful for practice.

**Results:**

A total of 3563 employees had complete data for analyses and 685 (19%) of them had long-term SA during 1-year follow-up. The final model 1 included age, gender, education, marital status, prior long-term SA, work pace, role clarity and learning opportunities. Discrimination between frequent absentees with and without long-term SA was significant (AUC 0.623; 95% CI 0.601–0.646), but not useful for practice. Model 2 showed comparable discrimination (AUC 0.624; 95% CI 0.596–0.651) with age, gender, education, marital status, prior long-term SA, burnout and work engagement as predictor variables. Differentiating by gender or sickness absence cause did not result in better discrimination.

**Conclusions:**

Both prediction models discriminated significantly between frequent absentees with and without long-term SA during 1-year follow-up, but have to be further developed for use in healthcare practice.

## Introduction

Frequent sickness absence (SA), that is three or more SA spells per year, is usually not considered an important problem because most of the time, frequent absentees are not long off work. However, previous research has shown that frequent SA is risk factor of long-term SA. Koopmans et al. (2008a) reported that 19% of the frequent absentees had long-term SA (i.e., 42 consecutive days or longer) in the first year of a 4-year follow-up study. During the whole 4-year follow-up period, 50% of the frequent absentees had long-term SA.

Few studies have investigated the potential risk factors of long-term SA among frequent absentees; we only found studies with employees with frequent SA as subgroups. Women with frequent SA were reported to have a higher risk of long-term SA than men with frequent SA (Koopmans et al. 2008a, b). Furthermore, frequent absentees with prior long-term SA were shown to have a higher risk of long-term SA during follow-up (Koopmans et al. 2008a, b; Stapelfeldt et al. 2014). Stapelfeldt et al. (2014) also investigated work characteristics as risk factors of long-term SA. Work pace, emotional demands, demands for hiding emotions, physical workload, influence, meaning of work, commitment to the workplace, role conflict and quality of leadership were dichotomized into favorable versus unfavorable and then summed into a score of unfavorable work factors. A higher score of unfavorable work factors was associated with an increased risk of long-term SA, but the authors did not specify the results for frequent absentees. Furthermore, the associations between individual work characteristics and long-term SA were not investigated.

Psychosocial work characteristics are known predictors of long-term SA (Stapelfeldt et al. 2014; Strømholm et al. 2015; Clausen et al. 2014; Borritz et al. 2010). Various theoretical models have been developed to explain the relationship between psychosocial work characteristics and SA. The Job Demands-Resources (JD-R) model is one of those theoretical models that allows a broad range of job demands (i.e., aspects of the job that require physical and/or psychological effort) and job resources (i.e., aspects of the job that are supportive for achieving goals and/or stimulate personal development) (Bakker and Demerouti 2007). The JD-R model posits a health impairment process, in which sustained high job demands lead to burnout and long-term SA (Clausen et al. 2012, 2014; Bakker et al. 2003; Slany et al. 2014). Sustained low job resources are associated with poor work engagement and both frequent and long-term SA (Clausen et al. 2014; Borritz et al. 2010; Slany et al. 2014; Schaufeli and Bakker 2004, 2009; Roelen et al. 2015; Rongen et al. 2015). There is some evidence that burnout lies on the pathway between job demands and long-term SA. Schaufeli et al. (2009) reported that an increase in job demands and a decrease in job resources predicted burnout, and that burnout predicted longer SA duration. Eriksson et al. (2008, 2011) described a burnout stair case, with job demands and job resources at the lower staircases, followed by burnout and SA at the highest staircase. We are not aware of earlier predictor model studies to predict long-term SA in a population of employees with frequent SA.

**Fig. 1:**
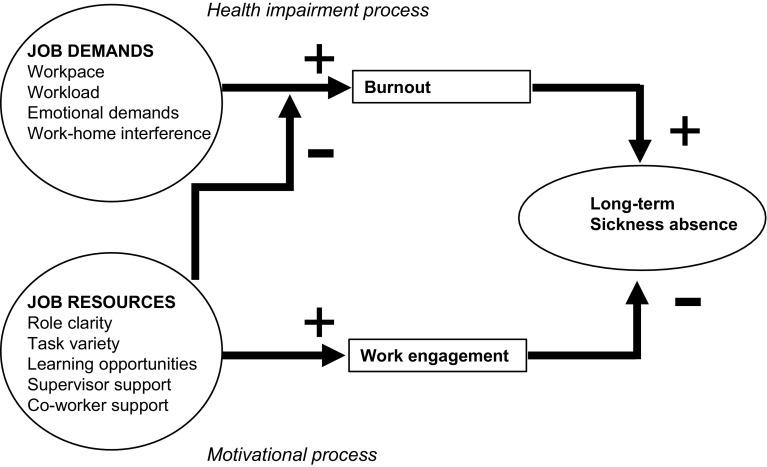
The job demands-resources (JD-R) model

The aim of the present study was to develop a prediction model for long-term SA among frequent absentees based on the predictor variables retrieved from the literature and the theoretical framework of the JD-R model, to enable timely prevention of long-term SA. If burnout and work engagement lie on the pathway between job demands and job resources on the one hand and long-term SA on the other, it would not be appropriate to include burnout and work engagement in a prediction model together with job demands and job resources (Fig. 1).

Therefore, we developed two prediction models: model 1, including job demands and job resources, not burnout nor work engagement and model 2 including burnout and work engagement, without job demands and job resources. We compared the models for their ability to predict long-term SA among frequent absentees. Job demands and job resources are likely to differ across work settings and it may be unfeasible to capture all potentially important job demands and job resources in the prediction model. Based on the JD-R model’s health impairment and motivational process, unmeasured job demands and job resources will also increase or reduce burnout and work engagement levels. Therefore, we hypothesized that a prediction model including burnout and work engagement instead of job demands and resources would better predict long-term SA among frequent absentees than a prediction model with job demands and job resources. For both models, we tested model performance of predicting long-term SA differentiating by gender and sickness absence cause.

## Methods

### Study setting and design

Surveillance of work and health is an important OHS task in The Netherlands. According to Dutch law, employers have to enable their personnel to participate in occupational health surveys every 4 years.

In the period between 2010 and 2013, 53,833 employees, working in companies across a wide range of economic sectors contracted by a large Dutch occupational health service, participated in occupational health surveys and completed questionnaires measuring health-related and work-related variables. The response rate varied across surveys between 40–60%. The survey participants (79% men) had a mean age of 45.1 [standard deviation (SD) 10.4] years and were working 38.1 (SD = 7.1) h per week for on average 15.7 (SD = 12.0) years in agriculture (3%), industry (71%), commercial services (14%), and public services (12%).

A total of 4204 (8%) employees participating in the occupational health surveys were eligible for the present study because they were frequent absentees in the sense that they had three or more SA spells in the year prior to the survey. The study was set up as an explorative cohort study based on a convenience sample in which baseline variables retrieved from the occupational health survey questionnaires were analysed against SA data recorded in the year following the survey. A total of 641 frequent absentees with missing responses on baseline predictor variables were excluded from the analyses. Consequently, complete cases analysis included the data of 3563 frequent absentees. The Medical Ethics Committee of the University Medical Center Groningen granted ethical clearance for this study (M12.116654).

### Outcome variable long-term SA

SA refers to a paid leave from work due to any (i.e., work-related as well as non-work-related) illness or injury. SA was recorded in an occupational health register from the day of reporting sick to the day of returning to work. In The Netherlands, SA has to be certified by an occupational physician (OP) if it lasts 42 days or longer. Therefore, we defined SA lasting ≥ 42 consecutive days as long-term SA, irrespective of cause. Long-term SA was obtained from the occupational health register in the year following the occupational health survey. Causes of long-term SA at follow-up were based on diagnoses of occupational physicians, translated into ICD-10 codes. Included were long-term SA due to mental and behavioral disorders (ICD-10 chapter V) and musculoskeletal and connective tissue diseases (ICD-10 chapter XIII).

### Predictor variables

Age (in years), gender (men; women), education (low = primary school and junior vocational education; medium = secondary general and senior vocational education; high = higher professional and academic education), and marital status (single; married; other, e.g., living with family) were obtained from the survey questionnaire.

Long-term SA (≥ 42 consecutive days) in the year prior to the occupational health survey was retrieved from the occupational health register and used for the predictor variable ‘prior long-term SA’ (no = 0, yes = 1).

Psychosocial work characteristics were measured with brief scales of the Questionnaire on the Experience and Evaluation of Work (QEEW) addressing work pace (5 items, Cronbach’s *α* = 0.87), cognitive demands (5 items, *α* = 0.83), emotional demands (3 items, *α* = 0.80), work–home interference (7 items, *α* = 0.88), role clarity (5 items, *α* = 0.85), task variety (6 items, *α* = 0.87), learning opportunities (4 items, *α* = 0.88), supervisor support (3 items, *α* = 0.90), and co-worker support (3 items, *α* = 0.89) (Van Veldhoven and Meijman 1994). In line with the JD-R model, work pace, cognitive demands, emotional demands and work–home interference were considered job demands. The job resources were role clarity, task variety, learning opportunities, and support from supervisors and co-workers.

The occupational health survey questionnaire measured burnout with the 15-item Dutch version of the Maslach Burnout Inventory-general survey (MBI-GS) covering emotional exhaustion (feelings of being emotionally overextended and exhausted by one’s work), cynicism (a feeling of distance and impersonal response towards recipients of one’s care or service) and personal accomplishment (feelings of competence and successful achievement in one’s work) (Bakker et al. 2002). All items had 7-point frequency response scales ranging from ‘never’ (= 0) to ‘always’ (= 6). Item scores were summed to a total MBI-GS score (*α* = 0.89) and divided by the number of items so that burnout scores ranged between 0 and 6. Higher scores represent higher levels of burnout.

Work engagement was measured with the 9-item Utrecht Work Engagement Scale (UWES) covering vigor (feeling strong and vigorous), dedication (enthusiasm about one’s job and feeling proud and inspired) and absorption (feeling flow when working) with 7-point frequency scales ranging from ‘never’ (= 0) to ‘always’ (= 6) (Schaufeli et al. 2006). Item scores were summed to a total UWES score (*α* = 0.94) and divided by the number of items so that work engagement scores ranged between 0 and 6. Higher scores represent higher levels of work engagement. In the literature, burnout and work engagement are described as closely related concepts (Demerouti et al. 2010; Mäkikangas et al. 2012). In our study, burnout and work engagement were correlated (Pearson correlation *r* = − 0.488), but not collinear.

### Statistical analyses

Statistical analyses were done with R for Windows (version 3.2.4) using the Regression Modelling Strategies (rms) package (version 5.1-1) (https://cran.r-project.org/web/packages/rms/rms.pdf). Age, job characteristics, burnout, and engagement were distributed normally. Student’s *t* tests for independent samples were used to determine differences in continuous baseline characteristics and Chi-square tests were used for the categorical variables. Gender, education, marital status, and prior long-term SA were included as categorical variables; age, work pace, workload, emotional demands, work–home interference, role clarity, task variety, learning opportunities, supervisor support co-worker support, burnout, and work engagement were included as continuous variables into logistic regression models with long-term SA (no = 0, yes = 1) in the year following the survey as outcome variable. Model 1 included age, gender, education, marital status, prior long-term SA, job demands, and job resources. Model 2 included age, gender, education, marital status, prior long-term SA, burnout and work engagement. Logistic regression analysis estimated odds ratios (OR) and related 95% confidence intervals (CI). The Wald-statistic is calculated using the formula (*B*/SE)^2^ where *B* is the regression coefficient and SE the standard error; higher Wald-statistics indicate stronger predictors. The prediction model was reduced by backward stepwise techniques, using Akaike’s information criterion (AIC) as a stopping rule.

The overall predictive performance of the final model was assessed by the Nagelkerke’s pseudo *R*^2^. Calibration refers to the agreement between predicted and observed risks and was investigated with the Hosmer–Lemeshow (H–L) goodness-of-fit test. H–L test *p* ≥ 0.05 indicates that the predicted risks do not deviate significantly from the observed risks, meaning that risk predictions are adequate. Discrimination refers to the ability of a prediction model to distinguish between frequent absentees with and without long-term SA during follow-up. Discrimination was investigated by receiver operating characteristic (ROC) analysis. The area under the ROC-curve (AUC) was used as measure of discrimination; AUC ≥ 0.75 represents discrimination useful for practice (Steyerberg 2009). All final models were stratified by gender. We also tested performance of the final models differentiating between sickness absence cause.

A prediction model will perform better in the subjects used to develop the model than in new subjects, a phenomenon known as overfitting. Overfitted prediction models are too optimistic in predicting outcomes for new subjects. Therefore, we internally validated the prediction models in 250 bootstrap samples to correct for over-optimistic predictions in new subjects.

## Results

The frequent absentees with complete data (*n* = 3563) were significantly older, higher educated, had significantly lower work engagement and significantly more frequently prior long-term than those excluded because of missing responses on the occupational health survey questionnaires (*n* = 641). Gender, marital status, prior SA frequency, job demands, job resources and burnout did not differ significantly between included and excluded participants (Table 1).

**Table 1:** Characteristics of the study population (*N* = 4204)

	Included participants (*n* = 3563)				Excluded participants^a^ (*n* = 641)				Analysis^b^
	Mean	SD^c^	*n*	%	Mean	SD	*n*	%	
Age (years)	44.2	10.9			43.0	10.7			*p* = 0.014
Gender									
Men			2399	67			398	62	*p* = 0.063
Women			1164	33			223	35	
Missing			0				20	3	
Education									
Low			800	22			174	27	*p* = 0.002
Medium			1618	45			248	39	
High			1145	32			176	27	
Missing			0				43	7	
Marital status									
Single			898	25			156	24	*p* = 0.609
Married			2524	71			403	63	
Other			141	4			26	4	
Missing			0				56	9	
Job demands (range 1–5)									
Work pace	2.7	0.9			2.7	0.9			*p* = 0.892
Cognitive demands	3.5	0.8			3.5	0.8			*p* = 0.266
Emotional demands	1.7	0.7			1.7	0.7			*p* = 0.842
Work–home interference	1.6	0.6			1.6	0.6			*p* = 0.932
Job resources (range 1–5)									
Role clarity	3.9	0.8			3.9	0.8			*p* = 0.953
Task variety	3.4	0.9			3.4	0.8			*p* = 0.675
Learning opportunities	2.8	1.0			2.8	1.0			*p* = 0.897
Supervisor support	3.5	1.1			3.5	1.1			*p* = 0.929
Co-worker support	3.8	0.9			3.8	0.9			*p* = 0.513
Burnout (range 0–6)	2.3	0.5			2.3	0.7			*p* = 0.119
Work engagement (range 0–6)	3.5	1.1			3.7	1.1			*p* = 0.001
Sickness absence spells in the year prior to the survey									
3			2031	57			337	53	*p* = 0.073
4			879	25			165	26	
5			353	10			66	10	
6			168	5			45	7	
> 6			132	4			28	4	
Long-term sickness absence in the year prior to the survey									
No			2648	74			543	85	*p* = 0.000
Yes			915	26			98	15	
Long-term sickness absence in the year following the survey									
No			2878	81			520	81	*p* = 0.870
Yes			685	19			121	19	

The table shows the characteristics of the participants in occupational health questionnaires who had three or more SA spells in the year prior to the survey. The table compares the baseline characteristics of included and excluded participants

^a^Exclusion because of missing responses on the baseline predictor variables

^b^Analysis of difference between included and excluded participants; Student’s *t* test for independent samples for continuous variables and Chi-square test for categorical variables

^c^*SD* standard deviation

During 1-year follow-up, 685 (19%) frequent absentees had long-term SA, predominantly due to musculoskeletal (*n* = 319; 47%) and mental (*n* = 256; 37%) disorders; 15 participants had both mental long-term SA and musculoskeletal long-term SA in the follow-up year. Other causes of long-term SA were cardiovascular (6%), gastro-intestinal (4%), neurological (4%), and various other disorders (2%).

### Performance of prediction model 1, with job demands and job resources

The full model 1 included 14 predictor variables. Based on the Wald-statistic, lower education, older age and female gender were the strongest predictors of long-term SA among frequent absentees. After backward stepwise reduction, 8 variables remained in the final model 1: age, gender, education, marital status, prior long-term SA, work pace, role clarity, and learning opportunities (Table 2).

**Table 2:** Prediction model on all-cause long-term sickness absence with job demands and job resources (model 1)

	Full model		Final model					
	All (*n* = 3563)		All (*n* = 3563)		Men (*n* = 2399)		Women (*n* = 1164)	
	Wald	OR (95% CI)	Wald	OR (95% CI)	Wald	OR (95% CI)	Wald	OR (95% CI)
Age	17.36	1.02 (1.01–1.03)	17.75	1.02 (1.01–1.03)	24.66	1.03 (1.02–1.04)	0.05	1.00 (0.99–1.02)
Gender	17.14		17.49		–		–	
Men		1		1		–		–
Women		1.49 (1.23–1.80)		1.49 (1.24–1.79)		–		–
Education	25.08		26.05		16.14		10.99	
Low		1		1		1		1
Medium		0.75 (0.61–0.92)		0.75 (0.61–0.92)		0.77 (0.60–0.99)		0.70 (0.48–1.03)
High		0.53 (0.41–0.68)		0.53 (0.41–0.67)		0.52 (0.38–0.72)		0.50 (0.33–0.76)
Marital status	5.90		6.09		6.81		1.18	
Single		1		1		1		1
Married		0.85 (0.70–1.03)		0.84 (0.69–1.02)		0.76 (0.59–0.98)		0.88 (0.65–1.19)
Other		0.54 (0.30–0.97)		0.54 (0.30–0.97)		0.47 (0.22–1.02)		0.66 (0.26–1.69)
Prior long-term SA	8.34		8.50		10.97		0.10	
No		1		1		1		1
Yes		1.32 (1.09–1.59)		1.32 (1.10–1.59)		1.48 (1.17–1.87)		1.05 (0.76–1.45)
Work pace	2.51	1.10 (0.98–1.24)	2.41	1.09 (0.98–1.21)	1.87	1.09 (0.96–1.24)	3.03	1.16 (0.98–1.36)
Cognitive demands	0.27	0.97 (0.85–1.10)						
Emotional demands	0.37	1.04 (0.91–1.20)						
Work-home interference	0.34	1.04 (0.90–1.21)						
Role clarity	2.54	0.90 (0.78–1.03)	2.67	0.90 (0.80–1.02)	0.25	1.04 (0.89–1.22)	12.23	0.70 (0.58–0.86)
Task variety	0.08	0.98 (0.86–1.12)						
Learning opportunities	2.42	0.91 (0.81–1.03)	3.96	0.91 (0.83-1.00)	3.73	0.89 (0.79-1.00)	1.57	0.91 (0.77–1.06)
Supervisor support	0.20	0.98 (0.88–1.08)						
Support co-workers	1.95	1.08 (0.97–1.21)						

The table shows Wald characteristics as indicator of predictor strength and the odds ratio (*OR*) and 95% confidence interval (*CI*) of associations between the health survey variables and all-cause long-term sickness absence (*SA*) for the full 14-predictor model and the final 8-predictor model obtained by backward stepwise statistical reduction. The final model was stratified by gender

**Fig. 2:**
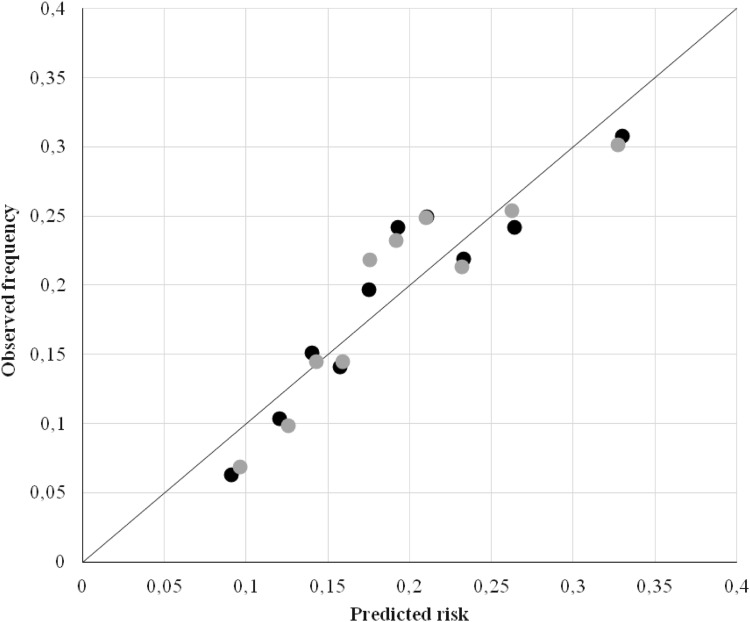
Calibration graph. The figure plots mean long-term SA risks predicted by the final 8-predictor model with job demand job resources model (black dots) and the final 7-predictor model with burnout and work engagement (grey dots) against observed frequencies  per decile of predicted risk; the diagonal indicates perfect calibration

The Nagelkerke’s pseudo *R*^2^ was 0.048, reflecting poor overall performance of the final logistic regression model. The H–L test *p* = 0.013 indicated that the risks predicted by the model deviated significantly from the observed risks of long-term SA, although inspection of the calibration plot showed no substantial deviations between predicted and observed long-term SA risks (Fig. 2). The full 14-predictor model had an AUC 0.625 (95% CI 0.599–0.654) and the final 8-predictor model 1 had AUC 0.623 (95% CI 0.601–0.646). Bootstrapping showed 4.8% over-optimism; the over-optimism adjusted AUC of the final model 1 was 0.615. Discrimination by the final model 1 did not differ between men (AUC 0.644; 95% CI 0.617–0.671) and women (AUC 0.622; 95% CI 0.583–0.660). Calibration was adequate for both men (H–L test *p* = 0.104) and women (H–L test *p* = 0.366). The Nagelkerke’s pseudo *R*^2^ was 0.064 for men and 0.043 for women. Age, gender, education, marital status, and prior long-term SA were strong predictors in the final model. When stratified, age, education, marital status and prior long-term SA were also strong predictors for men. For women, education and role clarity were strong predictors.

### Performance of prediction model 2, with burnout and work engagement

The full model 2 included 7 variables: age, gender, education, marital status, prior long-term SA, burnout, and work engagement. Again, lower education, older age, and female gender were the strongest predictors of long-term SA. After backward stepwise reduction, all 7 predictor variables remained in the final model 2 (Table 3).

**Table 3:** Prediction model on all-cause long-term sickness absence with burnout and work engagement (model 2)

	All (*n* = 3563)		Men (*n* = 2399)		Women (*n* = 1164)	
	Wald	OR (95% CI)	Wald	OR (95% CI)	Wald	OR (95% CI)
Age	16.98	1.02 (1.01–1.03)	24.73	1.03 (1.02–1.04)	0.06	1.00 (0.98–1.01)
Gender	16.96		–		–	
Men		1		–		–
Women		1.51 (1.26–1.81)		–		–
Education	27.17		19.14		9.53	
Low		1		1		1
Medium		0.72 (0.59–0.89)		0.75 (0.58–0.96)		0.71 (0.49–1.04)
High		0.53 (0.32–0.67)		0.50 (0.37–0.68)		0.54 (0.36–0.80)
Marital status	5.50		7.14		1.22	
Single		1		1		1
Married		0.86 (0.71–1.04)		0.75 (0.58–0.97)		0.91 (0.67–1.22)
Other		0.50 (0.28–0.91)		0.46 (0.21–0.99)		0.61 (0.24–1.56)
Prior long-term SA	8.96		10.69		0.00	
No		1		1		1
Yes		1.32 (1.10–1.57)		1.47 (1.17–1.86)		1.00 (0.73–1.38)
Burnout	6.55	1.22 (1.05–1.42)	5.87	1.26 (1.05–1.53)	1.01	1.15 (0.87–1.52)
Work engagement	1.78	0.95 (0.88–1.02)	0.30	0.97 (0.89–1.07)	2.69	0.89 (0.78–1.02)

The table shows Wald characteristics as indicator of predictor strength and the odds ratio (*OR*) and 95% confidence interval (*CI*) of associations between the health survey variables and all-cause long-term sickness absence. The model including men and women combined (all) concerns the full and final 7-predictor model. The model was stratified by gender

The overall predictive performance of the model was poor, with Nagelkerke’s pseudo *R*^2^ = 0.044. An H–L test *p* = 0.009 indicated miscalibration, although the calibration plot showed no substantial deviations between predicted and observed long-term SA risks (Fig. 2). The discriminative ability of the model was significant with AUC 0.624 (95% CI 0.596–0.651); after correction for 5.2% over-optimism, the AUC was 0.616. The final predictor model had AUC 0.646 (95% CI 0.619–0.673) for men, and AUC 0.583 (95% CI 0.544–0.622) for women. Calibration was adequate for the model with men and women with H–L test *p* = 0.436 and *p* = 0.632, respectively. The Nagelkerke’s pseudo *R*^2^ was 0.063 for men and 0.021 for women. For male frequent absentees, age, gender, education, marital status, prior long-term SA, and burnout were strong predictors of long-term SA, whereas for women education was the strongest predictor.

Table 4 shows the results of the final prediction models differentiated by sickness absence causes. When modelling only on participants with long-term SA due to mental disorders, discriminative ability was significant with AUC 0.635 (95% CI 0.599–0.670) for model 1 and AUC 0.610 (95% CI 0.574–0.646) for model 2. Discrimination was better, but still not useful for practice when modelling only on participants with long-term SA due to musculoskeletal disorders, with AUC 0.688 (95% CI 0.660–0.716) for model 1 and AUC 0.679 (95% CI 0.650–0.707) for model 2.

**Table 4:** Results per model for long-term SA due to different causes

	Nagelkerke’s pseudo *R*^2^	H–L test	AUC (95% CI)
Model 1			
All cause long-term SA	0.048	0.013	0.623 (0.601–0.646)
Mental long-term SA	0.040	0.712	0.635 (0.599–0.670)
Musculoskeletal long-term SA	0.079	0.866	0.688 (0.660–0.716)
Model 2			
All cause long-term SA	0.044	0.009	0.624 (0.596–0.651)
Mental long-term SA	0.030	0.815	0.610 (0.574–0.646)
Musculoskeletal long-term SA	0.071	0.730	0.679 (0.650–0.707)

The table presents prediction model performance measures differentiated by sickness absence cause; H–L test *p* ≥ 0.05 indicates adequate model calibration; the area under the receiver operating characteristic curve (*AUC*) reflects discrimination by the model between frequent absentees with and without long-term sickness absence during follow-up

## Discussion

We developed prediction models for the risk of long-term SA among frequent absentees using backward stepwise regression analysis. Final model 1 included age, gender, education, marital status, prior long-term SA, work pace, role clarity and learning opportunities. Discrimination by this model between frequent absentees with and without long-term SA during follow-up was significant, but not useful for practice. Model 2 included age, gender, education, marital status, prior long-term SA, burnout and work engagement. Discrimination between frequent absentees with and without long-term SA during follow-up was comparable to model 1. We hypothesized that it would be unfeasible to measure all potentially important job demands and job resources and therefore expected a better performance of model 2 as compared to model 1. Model 1 and model 2 showed comparable performance, even when stratifying the performance analysis by gender or when differentiating between long-term SA causes. Model 1 correctly identified frequent absentees with long-term SA during 1-year follow-up in 61.5% of the cases and model 2 in 61.6% of the cases. Although better than chance, discrimination of this magnitude is below the level recommended for practical use.

The poor discriminative ability is in line with previous research on prediction models for long-term SA. A prediction model including age, gender, education, self-rated health, mental health, prior long-term SA, work ability, emotional job demands, and recognition by the management correctly identified Danish employees at risk of long-term SA in 68% of the cases (Roelen et al. 2018). In a study on employees of an airline company, Boot et al. (2017) found that higher age, recent pregnancy, having a parking permit, having ‘aggravated working conditions’ (i.e., physical workload as a result of posture, lifting and abnormal working conditions) and prior SA correctly identified employees at risk of long-term SA in 73% of the cases. The better discriminative ability may be due to the fact that predictions were restricted to employees of one company, which enabled the investigators to include specific predictors, such as ‘having a parking permit’.

A recent study on prediction models including job demands and job resources showed poor discrimination between employees with and without long-term SA (Roelen et al. 2017). The prediction model, including psychological job demands, role conflict, harassment, role clarity, social support and fair leadership at the workplace, correctly identified nurses with long-term SA during 2-year follow-up in 56% of the cases. The explanation for the poor discriminative ability of this prediction model may be that the association of job demands and job resources with health outcomes differs across workplace settings; demands or resources that have a strong association with long-term SA in one workplace might be weakly or not associated with long-term SA in another workplace. Furthermore, there may be unknown job demands and job resources that are important predictors of long-term SA. Knowing we could not include all possible job demands and resources for all types of jobs and industries, we expected better predictions by the model including burnout and work engagement, because sustained high levels of both measured and unmeasured job demands will lead to burnout. Although the present study showed that higher burnout scores were associated with an increased long-term SA risk, discrimination between frequent absentees with and without long-term SA by the prediction model including burnout and work engagement was not better than discrimination by the model including job demands and job resources. Although both prediction models included psychosocial work factors, discrimination was not better for long-term SA due to mental disorders than for long-term SA due to musculoskeletal disorders. When stratifying the final models by gender, discrimination was comparable. These finding indicate that neither the model with job demands and job resources, nor the model with burnout and work engagement discriminates sufficiently between frequent absentees with and without long-term SA during 1-year follow-up. It is unlikely that longer follow-up periods improve the discrimination of baseline predictor models (Airaksinen et al. 2018). Although longer follow-up periods result in more events and higher statistical power, baseline predictor models predict outcomes most accurately on the short term (Melloh et al. 2012), particularly if predictor values change over time. Longitudinal prediction models with repeated measurements of predictor values over time may better discriminate between frequent absentees with and without long-term SA.

### Strengths and weaknesses

To our knowledge, this is the first cohort study investigating predictions of long-term SA among frequent absentees. Job demands, job resources, burnout and work engagement were all measured at baseline with reliable and valid scales and the analysis was based on the JD-R model as a theoretical framework (Van Veldhoven and Meijman 1994; Bakker et al. 2002; Schaufeli et al. 2006). Including burnout and work engagement could have improved the model (Borritz et al. 2010; Rongen et al. 2015), but due to the health impairment process described by the JD-R model, we decided to analyse job demands and job resources separately from burnout and work engagement. The data of the frequent absentees were obtained from a large population (*N* = 53,833) of employees who participated in occupational health surveys between 2010 and 2013. With 685 long-term SA episodes at follow-up and 14 variables in the full model we had almost 50 events per variable, which was sufficient for a robust backward regression analysis. Participants in health surveys may be healthier than non-participants (Froom et al. 1999). Healthy volunteer bias may have under-estimated associations between predictor variables and long-term SA, if healthy frequent absentees participated in health surveys more often than those with chronic health conditions. Although participants differed on some characteristics from those excluded at baseline, in most cases, the difference was small in absolute numbers except for prior long-term SA: 26% of the participants reported prior long-term SA as compared with 15% of those excluded at baseline. This may have resulted in overestimation of associations between prior long-term SA and long-term SA at follow-up. Selective participation may hamper the generalizability of the results.

The low Nagelkerke’s pseudo-*R*^2^ values indicate that important predictors of long-term SA among frequent absentees may be lacking from the prediction models. An earlier study (Slany et al. 2014) found indications that job demands and job resources predictive of long-term SA may differ between men and women. When stratifying our final models by gender, we also found that the predictor strength of several factors in women was different from men. Age, marital status and prior long-term SA were stronger predictors of long-term SA in male frequent absentees than in female frequent absentees. Role clarity was a strong predictor for women. However, the predictive performance of the models did not differ between men and women.

### Practical implications

The present study showed that education, age and gender were the strongest predictors of long-term SA among frequent absentees. We recommend health providers and managers to explore the causes of frequent SA in low educated, older and female frequent absentees as they are particularly at risk of long-term SA. With the current knowledge, this may be the best strategy for preventing long-term SA among frequent absentees. A prediction model for long-term SA would enable healthcare providers to better identify frequent absentees at increased risk of long-term SA and invite them for preventive consultations or refer them to interventions to reduce the risk of long-term SA. Prediction models including job demands and job resources or their effects in terms of burnout and work engagement proved to be better than chance, but have to be further developed for use in healthcare practice.

### Further research

The poor performance of the prediction models in the present study indicates that important predictors of long-term SA may be lacking from the models. More research is required to search for additional predictors of long-term SA among frequent absentees. Previous studies have included health-related predictors (Roelen et al. 2018; Boot et al. 2017; Laaksonen et al. 2011). Roskes et al. (2005) have reported that employees with chronic conditions have more frequent SA. Health-related variables may improve the predictions of long-term SA among frequent absentees. Furthermore, several studies have shown that work ability is a predictor of future long-term SA (Roelen et al. 2018; Reeuwijk et al. 2015; Schouten et al. 2015, 2016). We did not include work ability in the present study, because it was not measured in all occupational health surveys. Previous studies have shown that influence at work and quality of leadership predict long-term SA. Future studies could investigate if the prediction model for long-term SA among frequent absentees improves by adding work ability, health related variables such as self-rated health, influence at work or quality of leadership as a predictor variable. Furthermore, future studies should consider developing prediction models for men and women, as our present results show that predictors of long-term sickness absence differ between male and female frequent absentees. Age and prior long-term SA may not be included as predictor variables in the final prognostic model for long-term sickness absence in female frequent absentees.

## Conclusion

A prediction model including job demands and job resources and a prediction model including burnout and work engagement better than chance discriminated between frequent absentees with and without long-term SA during 1-year follow-up, but have to be further developed before using them to identify frequent absentees at risk of long-term SA and refer them to interventions aimed at preventing long-term SA.
